# Causal relationship between prostatic diseases and prostate cancer: a mendelian randomization study

**DOI:** 10.1186/s12885-024-12551-9

**Published:** 2024-06-27

**Authors:** Jiaguo Huang, Ji Sun, Kai Wang, Liying Zheng, Yi Fan, Biao Qian

**Affiliations:** 1 Department of Urology, Affiliated Xiaoshan Hospital, Hangzhou Normal University, Hangzhou, China; 2 Department of Urology, Affiliated Hangzhou First People’s Hospital, Zhejiang University School of Medicine, Hangzhou, China; 3 Department of Graduate, The First Affiliated Hospital of Gannan Medical College, Ganzhou, China; 4 Department of Urology, The First Affiliated Hospital of Gannan Medical College, Ganzhou, China

**Keywords:** Prostatitis, Benign prostatic hyperplasia, Prostate cancer, Risk factor, Mendelian randomization, GWAS

## Abstract

**Background:**

Although it is thought that prostatitis or benign prostatic hyperplasia (BPH) is related to prostate cancer (PCa), the underlying causal effects of these diseases are unclear.

**Methods:**

We assessed the causal relationship between prostatitis or BPH and PCa using a two-sample Mendelian randomization (MR) approach. The data utilized in this study were sourced from genome-wide association study. The association of genetic variants from cohorts of prostatitis or BPH and PCa patients was determined using inverse-variance weighted and MR Egger regression techniques. The direction of chance was determined using independent genetic variants with genome-wide significance (*P* < 5 × 10^–6^). The accuracy of the results was confirmed using sensitivity analyses.

**Results:**

MR analysis showed that BPH had a significant causal effect on PCa (Odds Ratio = 1.209, 95% Confidence Interval: 0.098–0.281, *P* = 5.079 × 10^− 5^) while prostatitis had no significant causal effect on PCa (*P* > 0.05). Additionally, the pleiotropic test and leave-one-out analysis showed the two-sample MR analyses were valid and reliable.

**Conclusions:**

This MR study supports that BPH has a positive causal effect on PCa, while genetically predicted prostatitis has no causal effect on PCa. Nonetheless, further studies should explore the underlying biochemical mechanism and potential therapeutic targets for the prevention of these diseases.

**Supplementary Information:**

The online version contains supplementary material available at 10.1186/s12885-024-12551-9.

## Introduction

Prostate cancer (PCa) is a common malignancy in men [1]. The incidence and mortality rates of PCa have increased in recent years due to the change in dietary structure and medical treatment [1]. PCa accounted for 7.3% of new male cancer cases worldwide in 2020, according to the Global Cancer Statistics from the United States [2, 3]. Besides family history/hereditary PCa [4, 5] and germline mutations [2], various exogenous and environmental factors, such as metabolic syndrome [6, 7], obesity [8, 9], and dietary factors, such as alcohol [10, 11], coffee [12], dairy [13], fat [14, 15], meat [16, 17], and vitamin D may cause PCa [18, 19].

Prostatitis and benign prostatic hyperplasia (BPH) are common prostatic diseases in men. Inflammation is closely related to the occurrence and progression of tumors [20, 21]. A retrospective study based on 746 176 participants showed that prostatitis is associated with an increased incidence rate of PCa. Furthermore, the study showed that the risk of PCa is higher in acute prostatitis than in chronic prostatitis [22]. Although many studies have assessed the relationship between BPH and the risk of PCa occurrence or death, this relationship is still unclear [23]. For instance, some studies have suggested that BPH increases [24, 25] or decreases [26] the risk of PCa, while others have found no association [27, 28]. However, these studies are mainly observational studies, which are more likely to be influenced by confounding factors.

Mendelian randomization (MR) is an epidemiological study design which limits the bias caused by common confounding and reverses causal relationships in observational studies. Genetic variants, robustly associated with a modifiable exposure, are used as instrumental variables (IVs) in MR to infer the causal relationship between the exposure and an outcome of interest [29, 30]. Two-sample MR has been widely used in various diseases due to the application of genome-wide association study (GWAS). This study aimed to evaluate the causal relationship between prostatitis or BPH and PCa based on the aggregated statistical data of large-scale GWAS using the two-sample MR analysis.

## Methods

Ethical approval was not needed since all data used had been previously published in the public database. Single nucleotide polymorphisms (SNPs) were used as IVs to investigate the causal relationship between prostatitis or BPH and prostate cancer. However, this approach requires the following three assumptions: (1) SNPs must have a strong association with prostatitis and BPH; (2) SNPs should not be affected by confounders that may impact the relationship between exposure and outcome; (3) SNPs should only impact the outcome through the exposure, and not through any other pathways.

To obtain a more reliable conclusion of the causal relationship, the largest public GWAS were searched for eligible summary-level data for each trait (Table 1). Specifically, summary statistics for prostatitis, BPH, and PCa were obtained from the Integrative Epidemiology Unit (IEU) Open GWAS project. In the original literature, diagnostic criteria and inclusion procedures are listed. Additionally, the participants were of European ancestry.

**Table 1:** Characteristics of prostatitis, BPH, and PCa GWAS cohorts

Phenotype	Participants	Population	Consortium	Years
Inflammatory diseases of prostate (prostatitis)	74 658	European	Not available	2021
Benign prostatic hyperplasia	463 010	European	MRC-IEU	2018
Malignant neoplasm of prostate	463 010	European	MRC-IEU	2018

*Note* BPH, Benign prostatic hyperplasia; GWAS, Genome-wide association study; IEU, Integrative Epidemiology Unit; MRC, Medical Research Council; PCa, Prostate cancer

The causal relationship between prostatitis or BPH and PCa was assessed using genetic instruments obtained from the MR-base database [31]. The SNPs associated with prostatitis and BPH were extracted at genome-wide significance (*p* < 5 × 10^–6^) using the stringent pairwise linkage disequilibrium (LD) r^2^ < 0.001 from a published GWAS meta-analysis. To make sure they were not related to any confounding factors (independence assumption), these SNPs were then checked in the database of human genotype–phenotype associations (http://www.phenoscanner.medschl.cam.ac.uk/). In addition, the IVs were evaluated based on R^2^ values and F statistic values to assess their correlation with exposure [32], as shown below, with the relevant variables noted.:

$${R}^{2}=2\times \left(1-MAF\right)\times \left(MAF\right)\times {\left(\frac{\beta }{SE\times \sqrt{N}}\right)}^{2}$$

$$F=\frac{N-k-1}{k} \times \frac{{R}^{2}}{1-{R}^{2}}$$

Note MAF, minor allele frequency; β, effect size; SE, standard error; N, sample size; k, number of SNPs.

### Statistical analyses

Several MR approaches (inverse variance weighted [IVW], weighted median, and MR-Egger) were used to determine MR estimates of prostatitis for PCa and BPH for PCa after harmonization of the effect alleles across the GWASs of prostatitis or BPH and PCa. Due to the different assumptions underlying horizontal pleiotropy, multiple approaches were employed. IVW meta-analysis of the wald ratio for individual SNPs was used as the main outcome. This IVW meta-analysis assumes that instruments can affect the outcome only through the exposure of interest and not by any alternative pathway [33]. MR-Egger and weighted median methods were used to complement IVW estimates since these approaches can provide more robust estimates in a broader set of scenarios but are less efficient (wider confidence intervals [CIs]) [34].

The heterogeneity for MR estimates can be severely violated even though sensitivity analysis is crucial to detect underlying pleiotropy in MR studies. In this study, heterogeneity markers (Cochran’s Q test *P* < 0.05) from the IVW approach indicated potential horizontal pleiotropy. The intercept obtained from the MR-Egger regression were used to represent directional pleiotropy (*P* < 0.05) [35]. Additionally, horizontal pleiotropy was assessed and corrected using MR-Pleiotropy Residual Sum and Outlier methods (MR-PRESSO) [34]. Three procedures are included in MR-PRESSO: (a) detection of horizontal pleiotropy; (b) correction for horizontal pleiotropy via outlier removal; (c) testing of significant differences in the causal estimates before and after correction for outliers. When the proportion of horizontal pleiotropy variants is less than 10%, MR-PRESSO exhibits lower bias and higher precision compared to IVW and MR-Egger [36]. To determine whether a single SNP was driving or biased the MR estimate, a leave-one-out analysis was also performed. Package Two Sample MR (version 0.4.25) and MR-PRESSO (version 1.0) in R (version 3.6.1) were used for analyses.

## Results

Eligible SNPs were selected as IVs to fit the three key assumptions after LD clumping (*p* < 5 × 10^–6^, LD r^2^ < 0.001), proxy SNP exploration, Phenoscanner database mining, and data harmonization. A total of 23 and 10 SNPs were for BPH and prostatitis, respectively. The F-statistics of more than the conventional value of 10 (F = 21.058 ~ 79.461) indicated a strong potential for these instruments, presenting a small possibility of weak instrumental variable bias. In Tables 2 and 3, we provide detailed information about IV treatments for BPH and prostatitis.

**Table 2:** Associations of SNPs with BPH

SNP	EA	NEA	EAF	BETA	SE	*P*	F
rs12027141	A	G	0.180	0.001	2.70 × 10^− 04^	1.90 × 10^− 06^	22.691
rs12131120	A	T	0.519	-0.001	2.08 × 10^− 04^	2.00 × 10^− 08^	31.483
rs4953671	T	G	0.329	-0.001	2.21 × 10^− 04^	1.30 × 10^− 06^	23.477
rs2556378	G	T	0.843	-0.002	2.83 × 10^− 04^	2.10 × 10^− 09^	35.868
rs11926963	T	C	0.218	0.001	2.55 × 10^− 04^	2.60 × 10^− 06^	22.062
rs13077048	T	A	0.427	0.001	2.11 × 10^− 04^	1.90 × 10^− 06^	22.709
rs35425714	A	C	0.282	0.001	2.37 × 10^− 04^	4.10 × 10^− 07^	25.665
rs1379553	G	A	0.208	-0.001	2.56 × 10^− 04^	3.10 × 10^− 08^	30.630
rs630231	T	C	0.904	0.002	3.52 × 10^− 04^	2.00 × 10^− 07^	26.996
rs380286	A	G	0.437	-0.002	2.08 × 10^− 04^	4.90 × 10^− 19^	79.461
rs9504961	T	C	0.682	-0.001	2.22 × 10^− 04^	8.70 × 10^− 07^	24.196
rs113360274	G	A	0.191	0.001	2.63 × 10^− 04^	1.70 × 10^− 06^	22.914
rs9348716	A	G	0.128	-0.002	3.11 × 10^− 04^	1.80 × 10^− 11^	45.128
rs2740817	T	C	0.227	0.001	2.47 × 10^− 04^	3.10 × 10^− 06^	21.741
rs10788160	A	G	0.251	0.002	2.38 × 10^− 04^	6.10 × 10^− 19^	79.023
rs12255539	A	G	0.191	0.002	2.63 × 10^− 04^	1.80 × 10^− 11^	45.140
rs4266963	C	T	0.758	-0.001	2.41 × 10^− 04^	6.90 × 10^− 10^	38.054
rs600231	G	A	0.314	-0.001	2.23 × 10^− 04^	3.30 × 10^− 07^	26.037
rs3116616	G	C	0.215	0.002	2.51 × 10^− 04^	4.20 × 10^− 10^	39.014
rs7162895	T	C	0.417	0.001	2.10 × 10^− 04^	3.30 × 10^− 06^	21.639
rs9958656	C	T	0.583	-0.001	2.10 × 10^− 04^	7.40 × 10^− 08^	28.956
rs11084596	C	T	0.384	-0.002	2.16 × 10^− 04^	5.60 × 10^− 13^	51.983
rs3213180	C	G	0.108	0.002	3.35 × 10^− 04^	2.80 × 10^− 07^	26.377

*Note* A, Adenine; BETA, effect size; BPH, benign prostatic hyperplasia; C, cytosine; EA, effect allele; EAF, effect allele frequency; G, guanine; NEA, other allele; SE, standard error; SNP, single nucleotide polymorphism; T, thymine

**Table 3:** Associations of SNPs with prostatitis

SNP	EA	NEA	EAF	BETA	SE	*P*	F
rs10915225	T	G	0.605	0.169	0.035	1.06 × 10^− 06^	23.853
rs4953907	C	T	0.103	-0.266	0.057	2.64 × 10^− 06^	22.081
rs9789699	G	A	0.308	0.175	0.037	2.10 × 10^− 06^	22.440
rs114884055	A	G	0.017	0.643	0.138	3.23 × 10^− 06^	21.674
rs76569337	C	T	0.204	0.233	0.043	4.21 × 10^− 08^	30.004
rs117901033	T	C	0.012	0.841	0.168	5.83 × 10^− 07^	24.970
rs79554384	T	C	0.067	0.317	0.069	4.40 × 10^− 06^	21.058
rs79165844	A	C	0.117	0.246	0.053	4.11 × 10^− 06^	21.204
rs35521406	G	A	0.192	0.216	0.043	6.18 × 10^− 07^	24.861
rs66617371	T	C	0.377	0.163	0.035	3.90 × 10^− 06^	21.364

*Note* A, adenine; BETA, effect size; C, cytosine; EA, effect allele; EAF, effect allele frequency; G, guanine; NEA, other allele; SE, standard error; SNP, single nucleotide polymorphism; T, thymine

### Causal effects of BPH on PCa

**Fig. 1:**
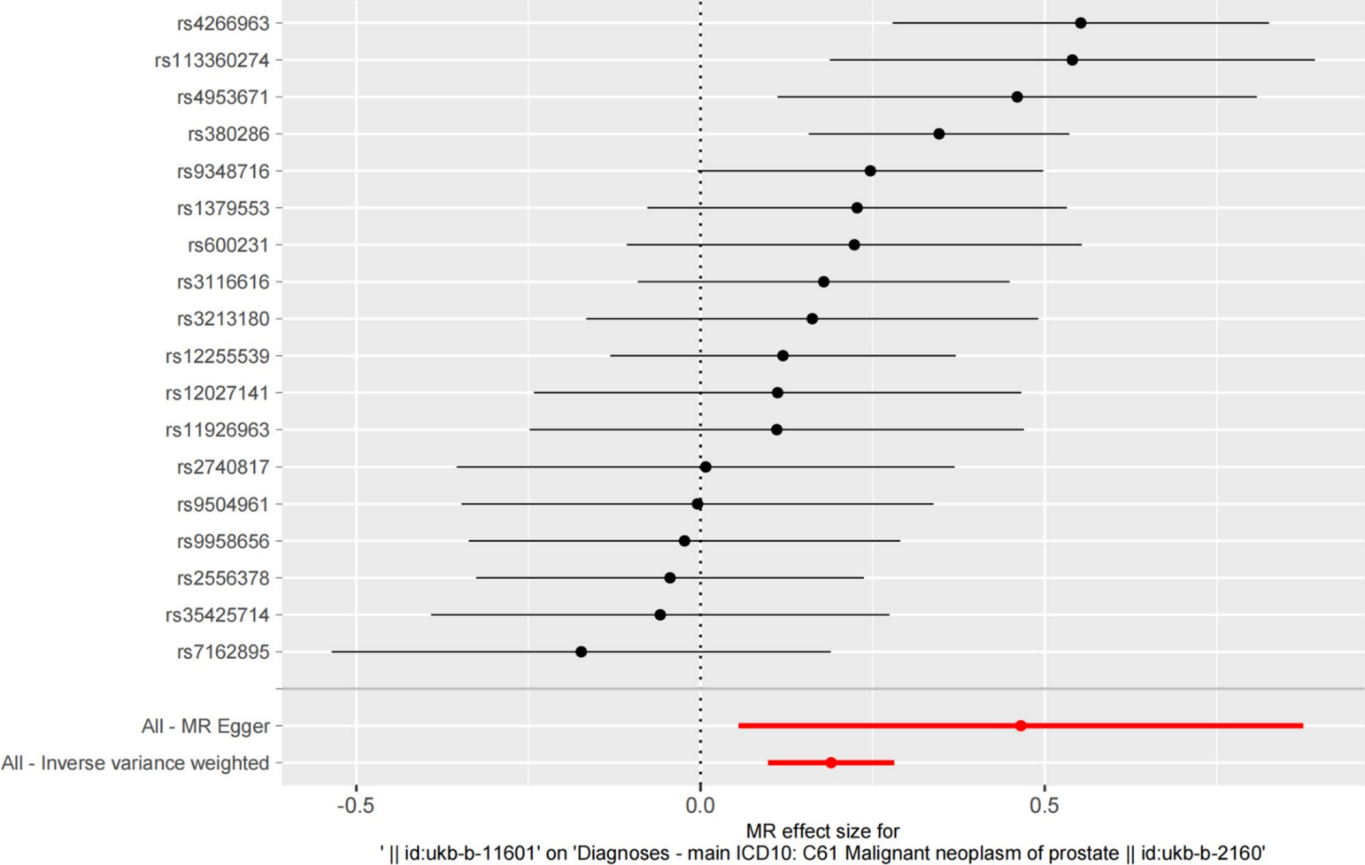
Forest plot for the causal effect of BPH on the risk of PCa. *Note* BPH, benign prostatic hyperplasia; MR, mendelian randomization; PCa, prostate cancer

There was significant heterogeneity in the Cochran’s Q test (*P* = 0.033), and thus the IVW method was applied with a random-effect model. BPH had a significant causal effect on PCa in IVW analysis (Odds Ratio [OR] = 1.209, 95% CI: 0.098–0.281, *P* = 5.079 × 10^− 5^), similar to MR-Egger results (Fig. 1). The black line segment in the figure represents the confidence intervals. The IVW and MR-Egger MR results are presented at the bottom of the figure. However, MR-Egger regression method did not detect any directional pleiotropy (intercept = -0.0004, *P* = 0.196). Although MR-PRESSO analysis did not reveal any outliers, heterogeneity existed (*P* = 0.033). The scatter plot and funnel plot were shown in Supplementary Figure S1-2. The slope of the straight line indicates the magnitude of the causal association. Leave-one-out sensitivity analysis results are shown in Supplementary Figure S3. The black line represents the deviation of the 95% CI corresponding to the estimate of the SNPs. The red line represents the estimated value of the IVW test. There was no difference with the final result after the removal of SNPs one by one.

### Causal effects of prostatitis on PCa

**Fig. 2:**
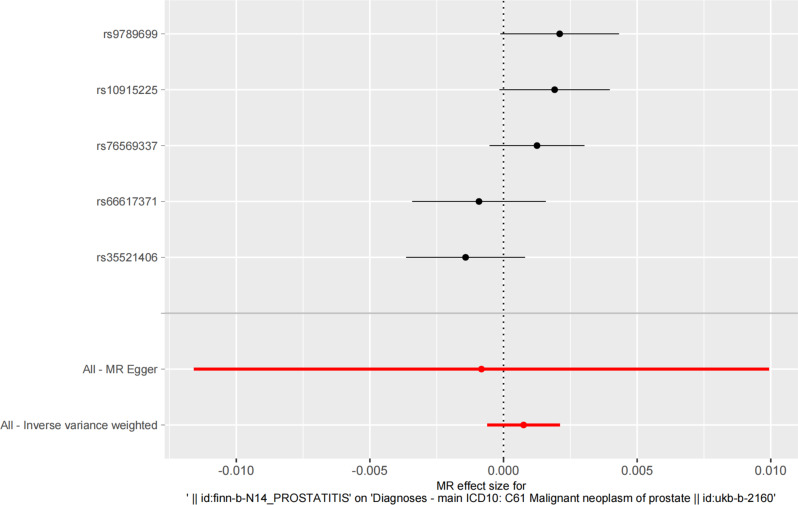
Forest plot for the causal effect of prostatitis on the risk of PCa. *Note* MR, mendelian randomization; PCa, prostate cancer

The fixed-effect IVW models were used in the main analysis after Cochran’s Q test due to the lack of heterogeneity. MR analysis demonstrated that genetically predicted prostatitis was not associated with PCa (OR = 1.001, 95% CI: -0.0002-0.002, *P* = 0.12, Fig. 2). The MR-Egger regression method did not identify horizontal pleiotropy (intercept = 0.0003, *P* = 0.79). Although MR-PRESSO analysis did not reveal any outliers, heterogeneity existed (*P* = 0.081). The scatter plot and funnel plot are shown in Supplementary Figure S4-5, while leave-one-out sensitivity analysis is shown in Supplementary Figure S6.

## Discussion

This study aimed to identify a potential causal relationship between prostatitis or BPH and PCa based on a two-sample MR approach, providing directions for further mechanistic investigations.

Some epidemiological studies have in recent years shown that BPH is associated with PCa, consistent with this study. Katharina Boehm et al. found that a BPH is associated with an increased risk of PCa based on a population-based case-control study (PROtEuS), especially for low-grade PCa [37]. Furthermore, a 27-year follow-up cohort study with 3 009 258 Danish men found that clinical BPH is associated with a two- to three-fold increased risk of PCa incidence and a two- to eight-fold increased risk of PCa mortality [25]. Although these studies matched age and ethnicity to minimize confounding effects, the inherent limitations of retrospective studies cannot be avoided.

In this study, results showed that BPH is a risk factor for PCa from a genetic perspective. Previous studies identified several potential mechanisms by which BPH exerts this effect. The common pathogenesis of BPH and PCa is age, genetics [38, 39], and the common pathogenesis of both, such as androgens, inflammation, obesity, metabolic syndrome, and diet [40–45]. Moreover, the process of seeking medical treatment for BPH often involves screening for PCa.

Although previous biological and epidemiological studies have shown that prostatitis is a risk factor for prostate cancer, the lack of cohort studies makes it difficult to conclude that there is a causal relationship between prostatitis and PCa. In this research, results showed that prostatitis did not influence the overall risk of PCa.

Inflammation, especially chronic inflammatory conditions, is associated with cancer development and progression. For instance, reflux esophagitis and virus hepatitis are associated with oesophageal cancer and hepatocellular carcinoma, respectively. Chronic inflammation induces carcinogenesis due to the local irritation associated with the regulation of the inflammatory cells and cytokine [46]. Both bacteria- and non-bacteria-related prostatitis (except for sexually transmitted diseases) are significantly associated with prostate cancer [47, 48]. Gyoohwan Jung et al. found that the incidence of PCa is significantly increased in 746 176 prostatitis patients (Hazard Ratio [HR] 2.99; 95% CI 2.89–3.09, *p* < 0.001). In that study, the HR for PCa was significantly higher in acute prostatitis than in chronic prostatitis (3.82 vs. 2.77) [22]. In an analysis of 167 autopsied prostates, Delongchamps et al. concluded chronic inflammation is frequently associated with BPH, but not with cancer [49]. Furthermore, chronic inflammatory infiltrations are located in the transitional zones instead of the peripheral zone where prostate cancer is usually diagnosed [50, 51]. However, the causality remains unclear.

However, it is difficult to confirm the causal relationship between prostatitis and PCa solely based on observational research since correlation studies cannot answer the question of causality. In summary, these findings should be carefully interpreted. Unlike most observational studies, we did not find a causal relationship between prostatitis and PCa, possibly due to use of different analytical methods.

This MR research has several advantages. First, to the best of our knowledge, this is the first study to evaluate the causal relationship between BPH and prostatitis on PCa using a dual sample MR analysis based on large-scale GWAS data. Compared with previous observational studies, MR analysis can effectively reduce potential biases, including confounding factors and reverse causal relationships, thereby enhancing causal inference. Second, the GWAS dataset for prostatitis, BPH, and PCa used is mainly based on populations of European ancestry and thus can minimize the impact of population stratification. Third, different estimation models and strict sensitivity analysis were used to ensure the reliability of the results.However, this study has some limitations. First, the study results do not represent a truly random population sample and are not applicable to other races since the data represent populations of European ancestry. Second, there may be some overlap in exposure and outcomes among participants, which can reduce data quality. Third, although various sensitivity analyses have been conducted to test the hypotheses of MR studies, it is also difficult to completely rule out the level pleiotropy of IVs. Pleiotropy broadly refers to SNPs being associated with effects in more than one trait. We can’t thoroughly rule out pleiotropic effects of the SNPs included in prostatitis or BPH that may confound the association, though we carefully selected the SNPs to avoid that. Finally, the current sample size of GWAS data is still large enough, and thus more GWAS data are needed to verify these findings.

## Conclusion

This MR study supports that BPH has a positive causal effect on PCa, while genetically predicted prostatitis has no causal effect on prostatitis. Nonetheless, more studies should explore the underlying biochemical mechanism and potential therapeutic targets for the prevention of BPH, prostatitis, and PCa.

### Electronic supplementary material

Below is the link to the electronic supplementary material.

Supplementary Material 1

Supplementary Material 2

Supplementary Material 3

Supplementary Material 4

Supplementary Material 5

Supplementary Material 6

## Data Availability

All data used in the current study are publicly available GWAS summary data (https://gwas.mrcieu.ac.uk/).

## Abbreviations

A: Adenine
BETA: Effect size
BPH: Benign prostatic hyperplasia
C: Cytosine
CI: Confidence interval
EA: Effect allele
EAF: Effect allele frequency
G: Guanine
GWAS: Genome-wide association study
HR: Hazard Ratio
IEU: Integrative Epidemiology Unit
IV: Instrumental variable
IVW: Inverse variance weighted
LD: Linkage disequilibrium
MAF: Minor allele frequency
MRC: Medical Research Council
MR: Mendelian randomization
NEA: Other allele
OR: Odds Ratio
PCa: Prostate cancer
SE: Standard error
SNP: Single nucleotide polymorphism
T: Thymine
